# Effect of Chemical Permeation Enhancers on Skin Permeability: *In silico* screening using Molecular Dynamics simulations

**DOI:** 10.1038/s41598-018-37900-0

**Published:** 2019-02-06

**Authors:** Rakesh Gupta, Balarama Sridhar Dwadasi, Beena Rai, Samir Mitragotri

**Affiliations:** 1 Physical Science Research Area, Tata Research Development and Design Centre, TCS Research, Tata Consultancy Services, 54B, Hadapsar Industrial Estate, Pune, 411013 India; 2 School of Engineering and Applied Sciences and Wyss Institute, Harvard University, 29 Oxford Street, Pierce 211, Cambridge, MA 02138, USA

## Abstract

Breaching of the skin barrier is essential for delivering active pharmaceutical ingredients (APIs) for pharmaceutical, dermatological and aesthetic applications. Chemical permeation enhancers (CPEs) are molecules that interact with the constituents of skin’s outermost and rate limiting layer stratum corneum (SC), and increase its permeability. Designing and testing of new CPEs is a resource intensive task, thus limiting the rate of discovery of new CPEs. *In-silico* screening of CPEs in a rigorous skin model could speed up the design of CPEs. In this study, we performed coarse grained (CG) molecule dynamics (MD) simulations of a multilayer skin lipid matrix in the presence of CPEs. The CPEs are chosen from different chemical functionalities including fatty acids, esters, and alcohols. A multi-layer *in-silico* skin model was developed. The CG parameters of permeation enhancers were also developed. Interactions of CPEs with SC lipids was studied *in silico* at three different CPE concentrations namely, 1% w/v, 3% w/v and 5% w/v. The partitioning and diffusion coefficients of CPEs in the SC lipids were found to be highly size- and structure-dependent and these dependencies are explained in terms of structural properties such as radial distribution function, area per lipid and order parameter. Finally, experimentally reported effects of CPEs on skin from the literature are compared with the simulation results. The trends obtained using simulations are in good agreement with the experimental measurements. The studies presented here validate the utility of *in-silico* models for designing, screening and testing of novel and effective CPEs.

## Introduction

Human skin provides an excellent barrier against the external harsh conditions, pathogens and other environmental threats. At the same time, it provides ample opportunities for transdermal drug administration owing to its large surface area and easy accessibility^1^. Transdermal drug delivery methods have certain advantages over conventional methods such as intravenous injections, oral ingestion and intramuscular injections^2,3^. For example, in case of oral administration, drugs have to pass through the gastro-intestinal (GI) tract, where they could be prone to degradation due to the harsh physicochemical environment^4^. Transdermal delivery eliminates drug exposure to the GI tract and first-pass metabolism, and ensures the sustained and controlled release of drugs^2–4^. Realizing the full potential of transdermal delivery, however, is limited by the protective barrier provided by the outermost layer of the skin known as stratum corneum (*SC*). The *SC* is about 15–20 µm in thickness and is comprised of keratin-rich corneocytes surrounded by the lipids^5,6^. The SC layer is arranged in a brick and mortar like structure where corneocytes occupy the majority of SC volume and the space between the corneocytes is filled with a lipid matrix which provides pathways for percutaneous absorption^6^. The SC is highly selective and only few molecules (small and relatively lipophilic) can pass through it. The SC is supported by viable epidermis, dermis and subcutaneous connective tissue, and these layers could potentially offer additional barriers to drug transport.

Effective breaching of the SC’s protective barrier is a major challenge in transdermal drug delivery. Hence, only a small number of transdermal formulations are commercially available^7^. Various ways have been proposed to breach the SC barrier and these methods are broadly classified as active and passive methods. The former class uses external energy sources such as iontophoresis^8^, sonophoresis^9^, microneedles^10^, electroporation^11^ to temporarily breach the skin barrier whereas the latter class uses chemical permeation enhancers and ionic liquids, among others^12,13^. Although, active methods offer quicker onset and have found applications in local anesthetic^14^, glucose monitoring^15^ and vaccination^16^, they pose certain limitations such as complexity and cost^17^. To date, several transdermal permeation enhancers have been studied^18,19^. More than 350 molecules have been shown to enhance the skin permeability via different mechanisms. These molecules include fatty acids and fatty alcohols, alcohols and glycols, terpenes, sulphoxides, laurocapram, pyrrolidones, surfactants, urea, among others^13,18,19^. However, very few of them have been successfully used in currently marketed transdermal products^19^. Hence, exploration of new chemicals that can safely improve skin permeability still remains an active area of transdermal research.

Several *in-vivo* and *in-vitro* studies have been carried out on animal or human skin to develop novel CPEs^18–26^. Efforts have also been made to use known CPEs to design their synergistic combinations. Specifically, a novel tool termed *in vitro* skin impedance guided high-throughput (INSIGHT) screening was developed and used to obtain synergistic mixtures of CPEs which could deliver macromolecular drugs, including heparin, luteinizing hormone releasing hormone (LHRH) and oligonucleotides, across the skin^20^. Attempts have also been made to study the mechanisms of action of CPEs and classify them based on their action rather than chemical identity^18,19,22^.

Design of CPEs based on first principles, in spite of its clear appeal, has been limited by the complexity of the enhancer-skin interactions. In the last decade, advances in computing hardware and development of efficient algorithms have encouraged researchers to use computer simulations for many drug delivery applications^27,28^. Researchers have also developed simple *in-silico* models of skin lipid matrix^29–31^ and successfully coupled them with macroscopic models^32^. These models have been validated with available experimental release profiles of drugs. In these reported studies, diffusion of molecules through the skin lipid matrix is obtained from molecular dynamics simulation and used as an input to the macroscopic (having both corneocyte and lipid matrix) model to obtain the release profile through the skin SC^32^. Recently, researchers have also explored computer simulations for the design of formulation using fullerenes for cosmetic application^33^, design of nanoparticle for drug delivery application^34^, and co-delivery mechanism of gold nanoparticles for protein delivery^35^.

In this study, we present the development of a multilayer *in-silico* multilayer skin lipid matrix model^34^ for testing the effect of CPEs on SC lipids. Long CG molecular dynamics (MD) simulations of multilayer skin lipid matrix model were carried out in the presence of CPEs at different concentrations (1, 3 and 5% w/v). Predictions of the simulations were compared to experimental measurements from the literature.

## System, Model, Parameters and Methods

### *in-silico* skin model

The corneocytes and skin lipids are organized in brick and mortar assembly inside the SC^6^. The lipid matrix is composed of various types of ceramides (CERs), fatty acids (FFAs) and cholesterol (CHOL)^36^. The CERs are classified based on the number and position of -OH groups and the degree of unsaturation present in the structure^37,38^. To date, 18 classes of the CERs have been discovered with over 300 to 1000 distinct CER derivatives^37,38^. In spite of the tremendous advances in computational capabilities in the last decade, simulating a multilayer skin lipid matrix with accurate CER and FFA distribution is still beyond the current technical feasibility. Here, we represent the ceramide family only by non-hydroxy sphingosine ceramide (CER-NS). Although it is a simple approximation, it has been validated in previous studies^33–35^. The skin permeation process is very slow and generally occurs at a ms-µs time scale depending upon the size, shape and surface chemistry of the molecule^39–41^. In order to model the heterogeneous mixture of CERs, FFAs, CHOL multi-layer with various chemical penetration enhancers at the realistic time and length scale, CG models^42,43^ were used. The CG parameters for ceramide (CER), free fatty acid (FFA) and CHOL were taken from our earlier work^33–35^. The parameters for the permeation enhancers molecules were derived from the atomistic models. In the MARTINI model, atoms were mapped to CG beads using mapping rules. Generally on an average four or three (for ring structures) heavy atoms were represented by a single bead. The parameterization of CPEs is discussed in section 2.2 in details.

**Figure 1:**
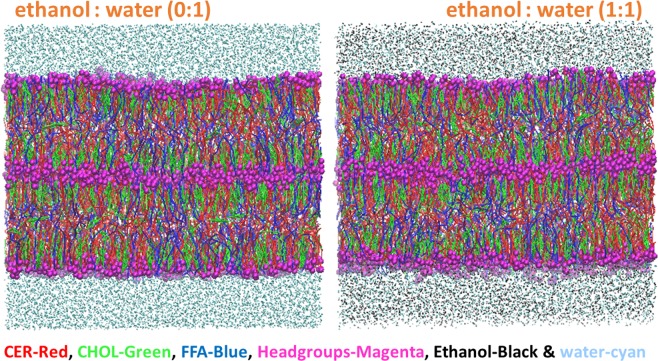
*In-silico* skin Model. The skin lipid layer model without ethanol (top-left) and with ethanol (top-right). Images were created using VMD software^60^. The ceramide, cholesterol, free fatty acid, lipid headgroups, water and ethanol are shown in red, green, blue, magenta, cyan and black color respectively. The headgroups are drawn in VDW style of VMD software.

The skin lipids are arranged in multiple lamellar domains. In order to simulate the realistic lamellar model, a multilayer skin lipid matrix model is used in this study. The CG structure of skin lipid bilayer (Fig. 1), equilibrated for 3 µs, was taken from our earlier work^34^. In the multilayer skin lipid matrix model, two lipid bilayers are kept next to each other and solvated with water across the top and bottom leaflet. In the experimental data used for comparison^20,22^ the formulation possessed 1.5% (w/v) CPE in 1:1 ethanol: phosphate buffered saline. The presence of phosphate ions in the buffer may potentially contribute to the experimental outcome which is not totally captured in the simulations. To mimic the experimental condition, half of the water in multilayer skin lipid matrix model was replaced with ethanol. The simulations were further run for 3 µs in NPT ensemble to obtain a structure (Fig. 1) that was used as an *in-silico* multilayer skin lipid matrix model for the testing. The simulation box possessed 936, 900, 936, 5760, 5760 CER, CHOL, FFA, water and ethanol molecules, respectively. The initial size of the lipid layer was 15.92 nm × 15.92 nm × 14.92 nm. Hence forward, *in-silico* multilayer skin lipid matrix model is referred as skin lipid layer.

### CG model of permeation enhancers

The CG model of CPEs was parameterized based on the MARTINI force field^42,43^. Mapping of the atomistic model on to a coarse grained model for each permeation enhancers is shown in Fig. S1 (see supporting information). The bead types were adopted from the MARTINI force field parameters^42,43^. The bonded parameters for CPEs were obtained by using the bond and the angle distributions obtained from the atomistic simulations as reference (Figs S2–S5). Parameters for the fatty acid enhancers were taken from the MARTINI force field^42,43^. In order to model the unsaturation of oleic acid, the angle was set to 120° and force constant of 55 kJ/mol/nm^2^ was used. More details on the parameters can be found in the supporting information.

In order to validate the CG models, the bulk phase density and *log P* (where *P* is the octanol water partition coefficient) were calculated and compared with the data obtained from the atomistic simulations and available experimental data^44–52^. We have used thermodynamic integration (TI) to calculate the free energies of solvation of molecules using their CG representation in water and octanol. These values were further used to calculate the water-octanol partition coefficients *log P*.

At first, atomistic bulk simulation of each permeation enhancer was carried out at 310 K in the NPT ensemble. For each test case, 300 molecules were randomly packed in a simulation box. All bonds were constrained using the LINCS algorithm. A time step of 2 fs was used for all simulations. A cutoff of 1.2 nm was used for van der Waals and electrostatic interactions. The long-range electrostatic interactions were computed using the particle mesh Ewald method. The simulation box was energy minimized and an NVT run of 10 ns was performed for equilibration. Finally, a 50 ns NPT run was performed and the last 20 ns data were used for the calculation of bond and angle distribution.

The CG bulk simulations of each CPE (with at-least 300 molecules of CPE) were performed for 10 ns to calculate the density under NPT conditions using a time step of 25 fs. The temperature was maintained at 310 K using a velocity-rescale thermostat and the pressure was maintained at 1 atm using Parrinello-Rahman barostat with time constants of 2 ps and 12 ps, respectively. Table 1 shows the comparison between the densities obtained from CG simulations (obtained density), all-atom simulations and the literature values. The CG simulations predicted the densities quite well.

**Table 1:** Comparison of Coarse Grained (CG) model with All Atomistic (AA) and literature data^a^.

Molecule	Density from literature	Density from atomistic simulation	Obtained density	*log P* from literature	Obtained *log P*
Geranic Acid	970^45^	987.36 (0.17)	955.06 (0.12)	3.70^45^	3.56 (0.07)
Geraniol	889^44^	864.09 (0.06)	870.8 (0.08)	3.56^44^	3.00 (0.09)
Isopropyl Palmitate	852^46^	884.74 (0.07)	857.91 (0.17)	8.16^46^	10.94 (0.10)
Monoolein	970^47^	946.29 (0.26)	964.65 (0.09)	6.40^47^	8.88 (0.10)
Limonene	841^48^	844.05 (0.07)	859.56 (0.06)	4.57^48^	5.75 (0.09)
N-Octyl pyrrolidone	920^49^	917.57 (0.07)	940.39 (0.71)	3.33^49^	3.65 (0.09)

^a^The data from literature is compiled from various sources^44–49^. The values in the brackets depict the standard error in the value. Density values are in kg/m^3^.

In the TI method, systems are divided into multiple windows where the interactions between the solute and solvent were gradually removed based on the decoupling parameter. Each window was simulated for 4 ns with a time step of 20 fs. Table 1 shows the *log P* obtained from CG simulation (obtained *log P*). The data for the CPEs, whose experimental data was not available in the open literature, was taken from the Estimation Program Interface (EPI) suite of the US EPA as given on chemspider website^44–49^. It is evident form Table 1 that our simulations capture the trend of the *log P* quite well, though some exceptions were found even after multiple trials. However, it should be noted that the literature values^44–52^ are also estimated (using the EPI suite generated values in chemspider website) and may have some error associated with them. As the values are much greater than one, we do not expect the qualitative trends of the partitioning to be significantly affected.

### Simulation Parameters

All simulations were carried out in NVT and NPT ensemble using the GROMACS MD package^53–55^. The pressure was controlled by Berendsen (equilibration run) and Parrinello-Rahman (production run) barostat with a time constant of 6 and 12 ps, respectively and the compressibility of 4.0 × 10^−5^ bar^−1^ with semi-isotropic coupling. The pressure was independently controlled in *XY* and *Z* directions to obtain a tensionless lipid layer. The temperature was controlled at ~310 K, using the Berendsen (equilibration run) and Nose-Hoover (production run) thermostat with a time constant of 2 ps. The LJ potentials were smoothly shifted to zero between a distance *r*_*shift*_ = 0.9 nm and the cutoff distance of 1.2 nm. Coulombic interactions were treated by a reaction-field with a cutoff of 1.1 nm and a relative electrostatic screening constant of 15. The pair list was updated at every 20 steps. The configuration was sampled at every 100 ps in the production run.

### Structural properties

#### Projected area on *XY* plane per lipid

In molecular dynamics simulation of a lipid bilayer, which has a normal along the Z direction, the area per lipid (APL) can be calculated using the following equation:

1 $$APL=2\frac{{L}_{x}{L}_{y}}{{N}_{lipid}}$$

where *L*_*x*_, *L*_*y*_ are the box lengths in X and Y direction, respectively and *N*_*lipid*_ is the total number of lipids in the bilayer.

#### Over all order parameter

The second rank order parameter for the bilayer, which has a normal in Z direction, could be defined as:

2 $${S}_{z}=\frac{1}{2}(3co{s}^{2}\theta -1)$$

where *θ* is the angle between the bonds and the bilayer normal. *S*_*z*_ = 1 corresponds to a perfect alignment with the bilayer normal, *S*_*z*_ = −0.5 anti-alignment, and *S*_*z*_ = 0 random orientation of the lipid chains.

The overall order parameter was calculated using following relationship:

3 $$\langle {\rm{S}}\rangle =\frac{{\sum }_{i=1}^{n}{S}_{z}(i)}{n}$$

where n is the number of beads in the alkyl chains in ceramide molecules and *Sz* is the order parameter for *i*^*t*h^ bead of ceramide alkyl chain.

## Results and Discussion

### Interactions of CPEs with skin lipid layer

The MD simulations of each permeation enhancer with skin lipid layer were carried out at three different concentrations (1% w/v, 3% w/v and 5% w/v). The permeation enhancers were randomly placed in the upper part of the multilayer model (near the headgroup of top layer). The system was energy-minimized using the steepest decent method. The minimized structure was subjected to a 200 ns NVT run by restricting the motion of the permeation enhancer molecules using position restraints. Later, the constraints were slowly released and the system was run for another 200 ns in NVT run followed by 250 ns in NPT equilibration. The final equilibrated structure was subjected to a 3 µs production run using the NPT ensemble. The configuration was sampled every 100 ps in the production run for the calculation of structural and thermodynamic properties.

**Figure 2:**
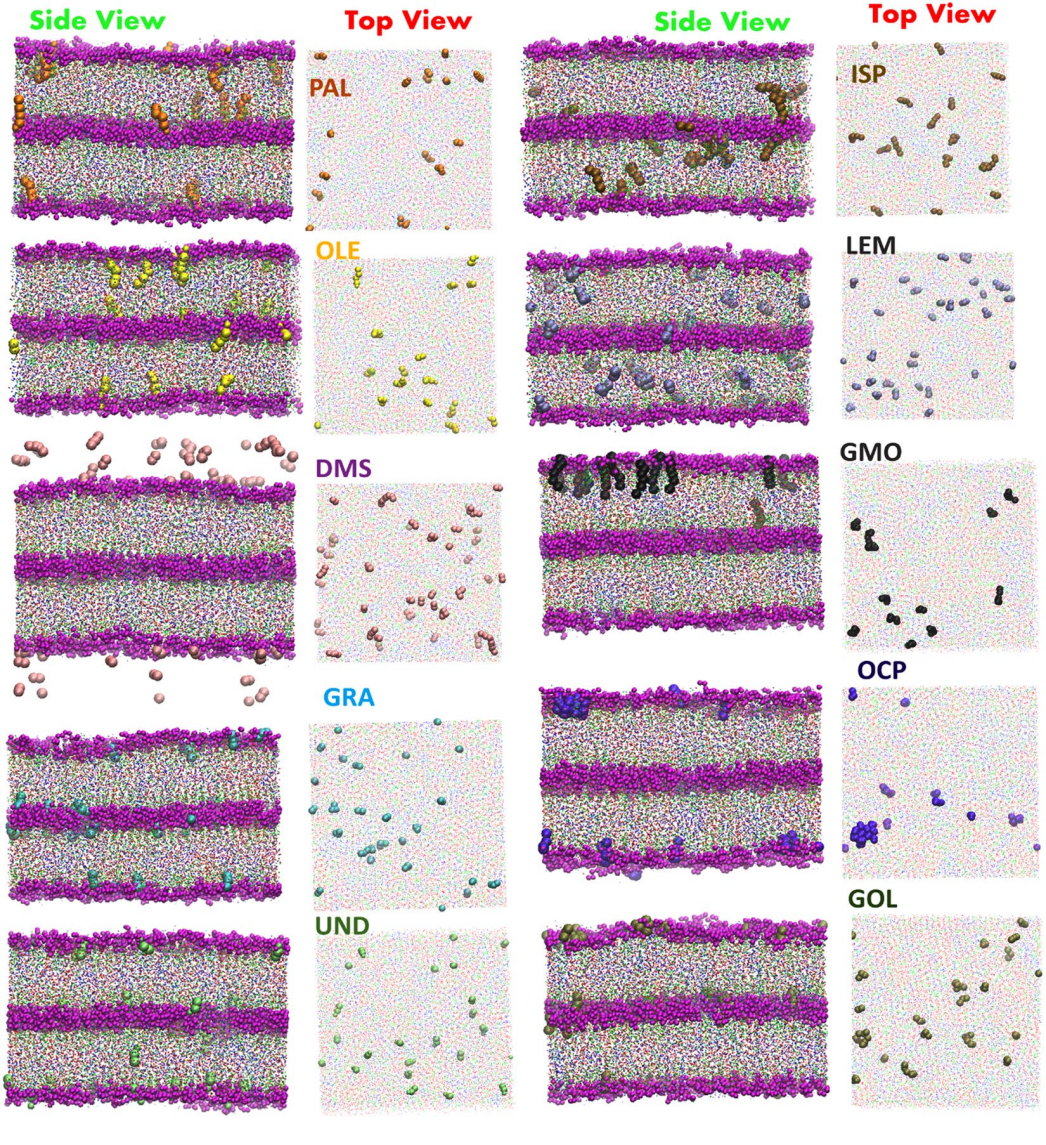
Interaction of CPEs with *in-silico* skin. Snapshots of the final configuration of the skin lipid layer interacting with chemical permeation enhancers (at 1%w/v concentration). Side view (shows permeation of CPEs) and top view (shows dispersion or agglomeration of CPEs inside the layers) are shown. All systems were run for 3 µs. The skin lipid constituent CER, CHOL and FFA are shown (in CPK form of VMD software) in red, green and blue colors, respectively. The solvent molecules (ethanol and water) are not shown in this figure. The permeation enhancers are shown in VDW form of VMD software. All snapshots were captured using VMD software^60^.

The permeation enhancers belong to several functional groups such as fatty acid, alcohols, esters, and terpenes, among others (Table S1). Figure 2 shows the final snapshots (at the end of 3 µs run) of each fatty acid system interacting with the skin lipid layer at a concentration of 1%w/v. The snapshots at 3%w/v and 5%w/v are shown in Figs S6 and S7 respectively. The mechanisms of interactions of each fatty acid with skin lipid layer are similar, first they partition from the upper (1:1: water: ethanol) layer to the upper leaflet of the lipid layer. Subsequently, they translocate in both lateral and normal directions of the lipid layer. Many of the fatty acid molecules also cross the first layer of the skin lipid layer and reach to the bottom of the skin lipid layer. Each enhancer was found to be well dispersed in the interior of the lipid layer (Top view Fig. 2).

Molecules such as Isopropyl palmiate (ISP), Octylpyrrolidone (OCP), Glyceryl monooleate (GMO) and Geraniol (GOL) partitioned completely from the upper solvent layer to the skin lipid layer (Fig. 2), whereas LEM partitioned partially and DMSO did not partition in any of the simulations. Permeation of ISP, LEM and GOL was similar to that of the fatty acids. While OCP and GMP partitioned into the lipids, they formed small clusters inside the skin lipid layer (Top view Fig. 2) hindering their motion in the normal direction, and very few molecules crossed the upper lipid layer. In our simulations, DMSO did not partition at any concentration. One reason could be the favorable condition provided by the solvent and second could be the concentration used here. In an earlier simulation study of DMSO with phospholipid bilayer, the partition and pore formation in the bilayer was achieved at a higher concentration (>26 mol %)^56^. Similarly, phase transition and partition of DMSO in CER bilayer occurred at very high concentration (>40 mol %)^57^. In current simulations, DMSO concentration is in the range of (<1 mol %) and hence could be a reason for lack of partitioning.

**Figure 3:**
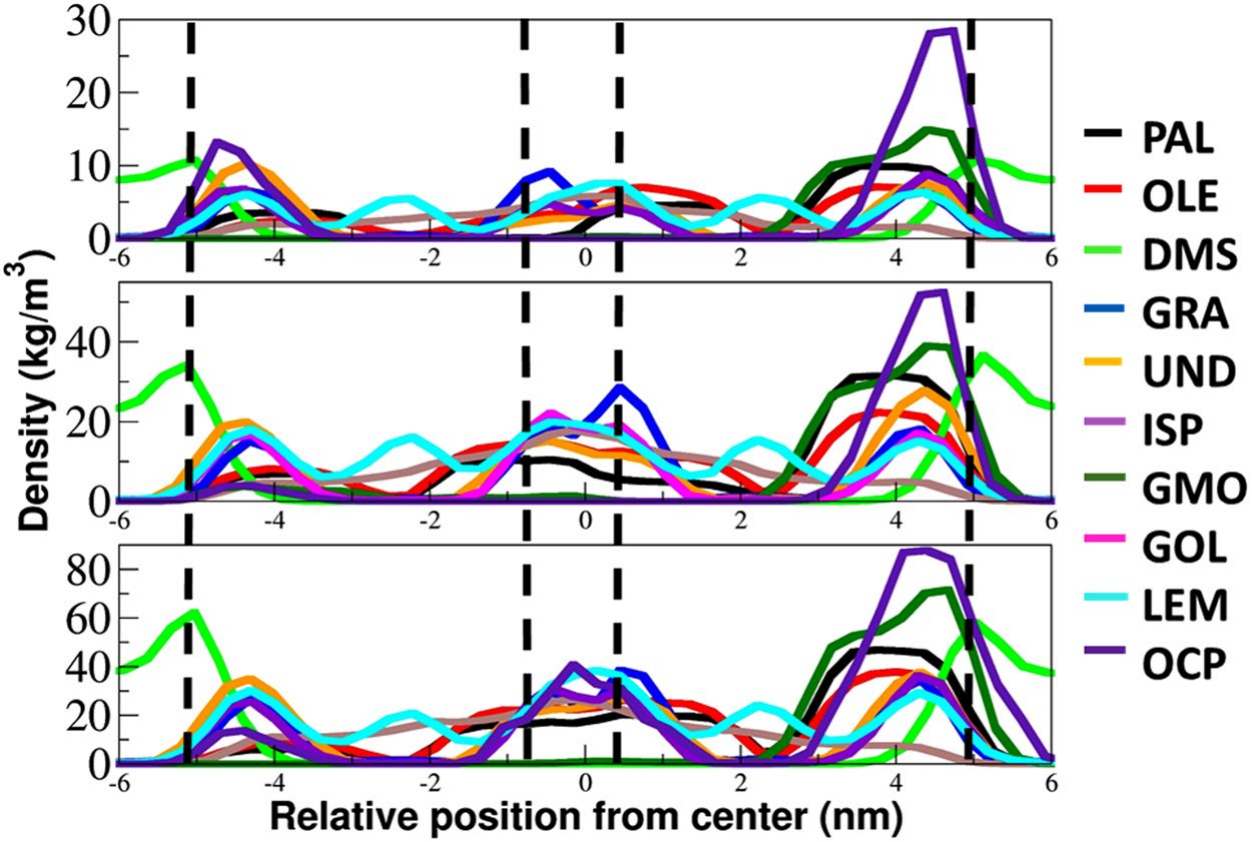
CPEs distribution in the skin lipid layer. The density distribution of each permeation enhancer along the lipid layer normal calculated in the last 1 µs of production run. The z = 0, represents the mid of the lipid layer. The dash black vertical lines indicate the average position of headgroups in each layer.

The density distributions of each penetration enhancer inside the lipid layer in different systems are shown in the Fig. 3. It is clear that each permeation enhancer, except DMSO, entered and crossed the skin lipid layer. The extent of permeation depends on the size, structure and interaction with the skin lipid layer constituents. Based on the hydrophobicity (as presented in terms of *log P*, Table 1), permeation enhancers partition from the solvent into the lipid layer and then move (both in lateral and normal directions) based on their mobility (diffusion coefficient).

**Figure 4:**
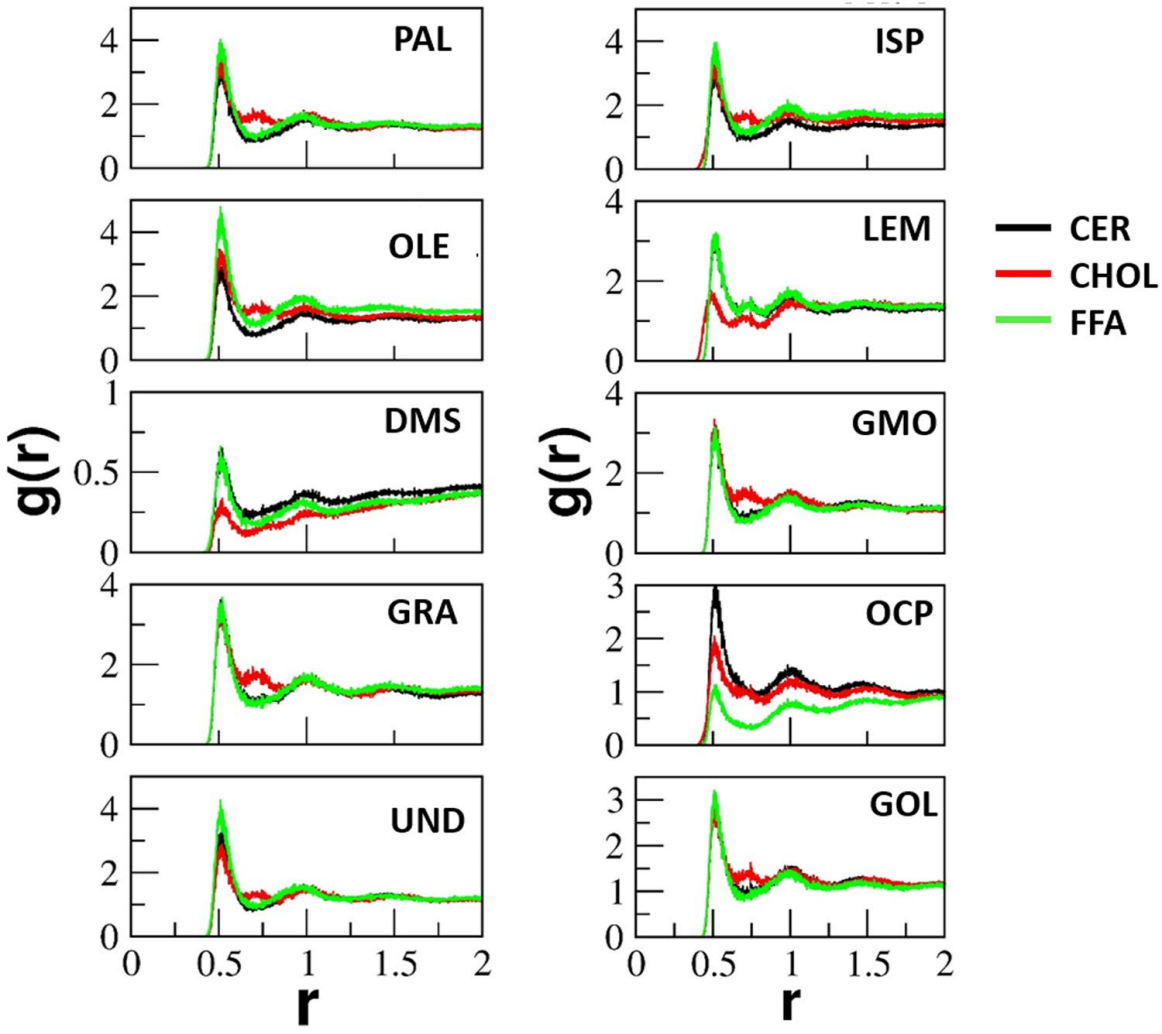
Interaction of CPEs with individual constituents of skin lipid layer. The radial distribution function g(r) of each permeation enhancer with the skin lipid constituents (at 1%w/v concentration), calculated in last 500 ns of production run. The peaks in g(r) profiles show the extent of the interaction between permeation enhancers and the particular lipid constituent.

The interactions of CPEs with skin lipid constituents were quantified in terms of radial distribution function g(r) and are shown in Fig. 4 (at 1%w/v concentration). The g(r) profiles for higher concentration 3%w/v and 5%w/v systems are provided in supporting information (Fig. S8). The radial distribution function was calculated using the GROMACS function g_rdf. The g(r) was calculated between the center of mass of individual lipids constituents (CER, FFA and CHOL) and each CPE. In case of fatty acids, long chain fatty acids PAL and OLE interacted heavily with each FFA, as can been seen by a clear peak position in g(r) profiles. On the other hand, small chain fatty acids UND and GRA interacted mostly with FFA and CHOL, as suggested by the dual peak of CHOL. This could be due to the reduced hydrophobicity of small fatty acid and large differences in chain length (UND chain length = 10 C united atom, GRA chain length = 8 C united atom) with that of FFA (chain length = 24 C united atoms) present in skin lipid layer. Other permeation enhancers interact with each of the lipid constituents. DMS did not partition inside the skin lipid layer hence g(r) values are lesser than 1.

### Structural Properties of Skin layer in the presence of CPEs

Each permeation enhancer molecule, other than DMSO, partitioned and moved to the top and subsequently bottom layer of the skin lipid layer (Fig. 2). These molecules significantly changed the lipid layer structural properties. We calculated two most important properties from the simulations namely, area per lipid (APL) and overall order parameter (<S>). The details of the calculation of these properties are given in section 2.4. The APL gives information about the extension of the lipid layer in the XY plane once the permeation enhancers are inside the lipid layer. The overall order parameter gives information regarding the orientation and disturbance in the packing of the lipid layer. Other two dynamics properties, percent permeated from one layer to another layer and diffusion coefficient of molecules inside the skin lipid layer were also calculated.

**Figure 5:**
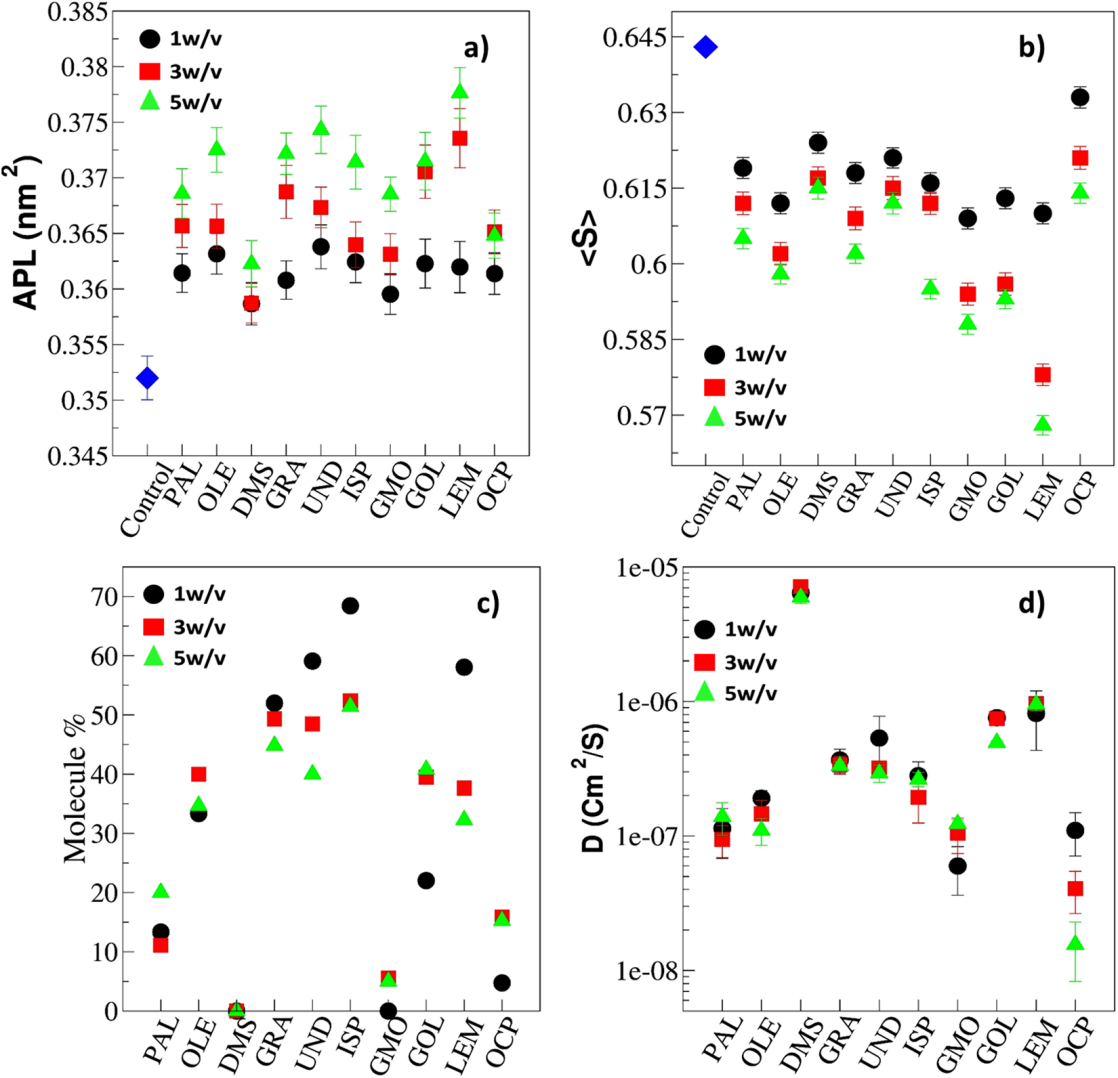
Effect of CPEs on structure of skin lipid layer. The structural properties: (**a**) area per lipid (APL), (**b**) overall order parameter (<S>) and dynamics properties, (**c**) percentage of molecule permeation from the top to the bottom layer (molecule %), and (**d**) diffusion coefficient of permeation enhancers in the skin lipid layer. The properties are calculated in the last 1 µs of production run. Control indicates the skin lipid layer system without permeation enhancer.

Figure 5a–d show the structural properties APL and <S> as well as the dynamics properties percent permeation and diffusion coefficients of each system at different concentrations. The percent permeation of CPEs is the fraction of total CPE molecules placed initially in the upper reservoir that crossed the upper leaflet and reached to the bottom leaflet. Due to periodic boundary conditions, in some cases few molecules moved from the upper reservoir to the bottom reservoir, these molecules were excluded from the calculation of percent permeation. The trajectory of each molecule was visualized and molecules that crossed the upper leaflet were counted manually. The diffusion coefficient was calculated from the mean square displacement determined using g_msd utility of GROMACS (see Fig. S9). There is qualitative inverse correlation between APL and overall order parameter. It is clear that at higher concentrations, more molecules permeated into the lipid layer and they induced expansion of the lipid layer in the lateral direction. At the same time, increased CPE molecules present in the lipids reduced the order and the order parameter.

In case of fatty acids, the oleic acid system led to lowest <S> and UND led to highest APL at a given concentration. The increased APL for UND system originates from the permeation of larger number of molecules (Fig. 5c). The OLE system has a lower order parameter because of its unsaturation, which provides a kink in the skin lipid layer. All fatty acid molecules remained dispersed in the skin lipid layer (Top view Fig. 2), as their diffusion coefficient did not change significantly with the concentration (Fig. 5d). The smaller sized UND and GRA exhibited a high diffusion coefficient as compared to PAL and OLE, which resulted in their higher molecular permeation. An interesting point to be noted is that PAL and OLE exhibited a similar order of diffusion coefficient, but OLE exhibited higher permeation. This is because oleic acid significantly disturbs the packing due to its structure. The packing disturbance could be related to the order parameter. Based on the simulations, the trend for disturbing the packing by fatty acids should be in the following order OLE > PAL > GRA > UND. Our observations of permeation enhancing mechanism of fatty acid are well in line with the experimental findings reported in the literature^21–23^.

Kim *et al*.^21^ have performed permeation experiments of diclofenac in the presence of various fatty acids on rat-skin and showed that palmitic acid and oleic acid were the best permeation enhancers in the saturated and unsaturated category, respectively. Our simulations predicted that oleic acid worked as the best permeation enhancer among all fatty acids studied. Karande *et al*.^22^ have performed experiments on porcine skin and measured skin conductivity before and after the application of fatty acid permeation enhancers. The enhancement in skin conductivity was related with the enhancement in molecular flux^58^. The experimental ER values reported for OLE, PAL and UND were 30.01, 18.87 and 3.66, respectively. Ibrahim *et al*.^23^ studied the effects of fatty acids that are commonly present in cosmetic and in topical formulations on permeation enhancement across the human epidermal membrane (HEM). The flux enhancement ratio was in the order of OLE > PAL > UND. Kezutyte *et al*.^59^ performed permeation experiment of lipophilic model drug tolnaftate in the presence of oleic, linoleic, lauric and capric acids into human skin using time-of-flight secondary ion mass spectrometry (TOF-SIMS) imaging. It was shown that the flux of the drug was maximum (1.87 times of control experiment) in the presence of oleic acid.

DMSO exhibited minimum APL values and diffusion coefficients were found to be an order of magnitude higher for a given concentration as it did not partition in the lipid layer. However, surprisingly, the overall order parameter was not the maximum for the DMSO. The OCP system exhibited maximum <S> for a given concentration, the reason could be the agglomeration of OCP (Top view Fig. 2) in the skin lipid layer after partitioning. The cluster creates local changes in the tail order parameter, while if the molecules were well dispersed (like in the case of other enhancers) they disturbed the packing throughout the lipid layer. The diffusion coefficient values also decreased (due to agglomeration, Fig. 2) with an increase in the concentration of OCP. GMO also formed small clusters, but affected the order parameter significantly due to its structure. The GMO molecules did not cross the lipid layer significantly. Small molecules such as LEM and ISP crossed the lipid layer significantly (as can be seen from the percentage molecule permeate and diffusion coefficient) but did not create much disordering in the skin lipid layer as compared to GMO.

Overall, based upon the above four parameters and observation (Table 2), it could be concluded that small hydrophobic molecules partition well into the skin lipid layer and do not agglomerate. On the other hand, bigger hydrophobic molecules partition well and disturb the lipid layer packing significantly, but they sometime form small clusters and limit permeation by the diffusion rate.

**Table 2:** The structural and physical properties of each permeation enhancer used in simulation and the observation obtained after the production run.

Name	Functional group	ER^22^	*log P* (simulation)	Observations
Oleic Acid (OLE)	Acid	30.01	—	• Complete partitioning from solvent to the top layer of skin lipid layer • Dispersed in lipid layer and many molecules are crossing lipid layers
Palmitic Acid (PLA)	Acid	18.81	—	• Complete partitioning from solvent to the top layer of skin lipid layer • Dispersed in lipid layer and many molecules are crossing lipid layers
Geranic Acid (GRA)	Acid	—	3.56	• Complete partitioning from solvent to the top layer of skin lipid layer • Dispersed in lipid layer and many molecules are crossing lipid layers
Undecanoic acid (UND)	Acid	13.66	—	• Complete partitioning from solvent to the top layer of skin lipid layer • Dispersed in lipid layer and many molecules are crossing lipid layers
DMSO (DMS)	DMSO	15.13	—	• No Partitioning from solvent to the top layer of skin lipid layer • Dispersed in solvent (water: ethanol) layer and no crossing of lipid layer
Geraniol (GOL)	Unsaturated alcohol	32.74	3	• Partitioning from solvent to the top layer of skin lipid layer • Dispersed in lipid layer and crossing lipid layers
Glycerylmonooleate (GMO)	Glyceryl ester	33.23	8.88	• Complete partitioning from solvent to the top layer of skin lipid layer • Small clusters are forming in the lipid bilayer and very few molecules crosses layer
Isopropyl palmiate (ISP)	ester	27.11	10.94	• Complete partitioning from solvent to the top layer of skin lipid layer • Dispersed in lipid layer and crossing lipid bilayer
Limonene (LEM)	Unsaturated monoterpene	22.32	5.75	• Partitioning from solvent to the top layer of skin lipid layer • Dispersed in lipid layer and crossing lipid bilayer
Octyl pyrrolidone (OCP)	Pyrrolidone	7.65	3.65	• Partitioning from solvent to the top layer of skin lipid layer • Agglomerating in the skin lipid bilayer and very few molecules crossed the layer at high concentration

### *In-silico* screening

Based on the conclusions made in section 3.2, the overall order parameter could be used for quantifying the structural changes induced by CPEs in the skin lipid layer. Experimental evaluation of overall order parameter is extremely challenging. Hence, indirect comparisons of predicted order parameters with experimental measurements are necessary. Karande *et al*.^22^ have reported on the effect of several CPEs on porcine skin which was quantified as the conductivity enhancement ratio (ER) defined as skin conductivity at the end of 24 h normalized by that at time zero. Since conductivity is a measure of ion mobility in the skin which is limited by diffusion across the lipids, the enhancement ratio is a measure of CPE-induced lipid disruption.

**Figure 6:**
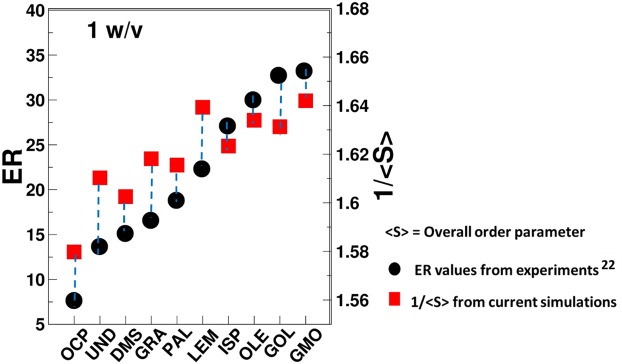
*In-silico* testing validation. Comparison of experimentally measured enhancement ratio (ER) and the calculated (1/<S>) from simulations. Both properties are plotted on different axes due to the differences in the magnitudes of each property.

In simulations, overall order parameter also reflects disruption of the lipids. Specifically, the order parameter is inversely proportional to the extent of lipid disruption. Figure 6 shows the comparison of ER and reciprocal of overall order parameter (1/<S>) for each CPE at 1%w/v. Comparisons of ER and 1/<S> for higher concentration 3%w/v and 5%w/v are provided in supporting information (Fig. S10). Experimental ER and simulated 1/<S> exhibited a good correlation. For example, ER values of fatty acids showed the trend of OLE > PLE > UND, and a similar trend was observed in simulations. Another significant trend was observed for OCP/GMO, which exhibited the lowest/highest ER and the lowest/highest 1/<S> value for each concentration. GMO exhibited highest perturbation of lipids and it interacted extensively with all three constituents of the skin lipids (as shown through radial distribution function in Fig. 4). OCP also exhibited affinity towards CER and CHOL, but due to agglomeration (Fig. 2) and less diffusion (Fig. 5d), it did not significantly perturb the packing, hence exhibited a lower permeation enhancing effect.

The values for DMSO cannot be compared directly with the experiments, since it did not partition inside the skin lipid layer, however it changed the lipid layer orientation by interacting with the headgroup beads of CER and FFA. Overall, an effective permeation enhancer should partition from the solvent into the lipid layer, and also interact with the skin lipid layer constituents and disturb the packing.

The *in-silico* model presented here reasonably captures the experimentally observed trends and could be used for future testing purposes. Some challenges remain which need to be further explored. First, the model only used single type of CER, which is a simplistic representation of skin lipid layer. We are currently developing parameters for other CERs which will further enhance the model in future. Second, the model only has a lipid matrix, but, some CPEs first partition into corneocytes and then into the lipid layer. Studying such CPEs, with current model, may not show partitioning at all. For example, DMSO was not able to partition because of its hydrophilicity and low concentration. Simulations of corneocytes is a challenging task. However, a combination of a micro and macro scale model that incorporate corneocytes may pave the way. Also note that the toxicity of CPEs could not be captured using current model since it does not incorporate biological elements required for assessment of toxicity.

## Conclusions

The use of computer simulations for screening of permeation enhancers is shown by performing multiple and long CG MD simulations on an *in-silico* multilayer skin model with various CPEs. The permeation mechanism was found to be highly structure- and size-dependent. To cross the skin lipid layer, the permeation enhancer has to partition from the donor solution into lipid layer. This partitioning of CPEs into the skin lipid layer depends significantly on their hydrophobicity (*log P*). Once the CPEs partition into the lipid layer, their movement inside the skin lipid layer depends highly on their interactions with lipid constituents and themselves. Based on these factors, several peculiar phenomena such as smaller cluster formation, agglomeration and dispersion were observed in our simulations. The CPEs changed the lipid layer ordering. The overall lipid order parameter compared favorably with experimental ER. The *in-silico* model could be used for screening CPEs. For a molecule to serve as a better permeation enhancer, it should not only be able to partition effectively from the solvent/donor solution into the SC lipids, but also interact with the skin lipid layer constituents to create transient structural perturbations to increase permeability. Future studies should focus on increasing the complexity of the model by including other ceramides and corneocytes. The advancement in computing architecture and development of new CG models will enable such studies.

## Supplementary information

Supporting information

## Data Availability

All data generated during this study are included in this published article and it’s Supporting Information.
