# Supplementary material for: Effect of Chemical Permeation Enhancers on Skin Permeability: In silico screening using Molecular Dynamics simulations
Source: Sci Rep. 2019 Feb 6;9:1456. doi: 10.1038/s41598-018-37900-0 (PMC6365548; doi:10.1038/s41598-018-37900-0)
Supplement: Supplementary file 1 — Supporting information [file 41598_2018_37900_MOESM1_ESM.pdf]

Effect of Chemical Permeation Enhancers on Skin Permeability: In silico screening using Molecular Dynamics simulations

Rakesh Gupta[1,*], **Sridhar Dwadasi**[1], **Beena Rai**[1] and **Samir Mitragotri**[2]

Physical Science Research Area, Tata Research Development and Design Centre, TCS Research, Tata Consultancy Services, 54B, Hadapsar Industrial Estate, Pune – 411013, India

[2]School of Engineering and Applied Sciences and Wyss Institute, Harvard University

*Corresponding author: gupta.rakesh2@tcs.com

Fax : 91-20-66086399

Phone : 91-20-66086203

Supporting Information

S1. Systems used in simulations

Table S1. The details of the permeation enhancers and number of the enhancers used in each simulation.

Name	Functional group	Number of Permeation enhancers (1 w/v)	Number of Permeation enhancers (3 w/v)	Number of Permeation enhancers (5 w/v)
Oleic Acid (OLE)	Acid	15	45	75
Palmitic Acid (PLA)	Acid	16	48	80
Geranic Acid (GRA)	Acid	25	75	125
Undecanoic acid (UND)	Acid	22	66	110
DMSO (DMS)	DMSO	57	171	285
Geraniol (GOL)	Unsaturated alcohol	27	81	135
Glyceryl monooleate (GMO)	Glyceryl ester	12	36	60
Isopropyl palmiate (ISP)	ester	14	42	70
Limonene (LEM)	Unsaturated monoterpene	31	93	155
Octylpyrrolidone (OCT)	Pyrrolidone	21	63	105

S2. Parameterization of CPEs

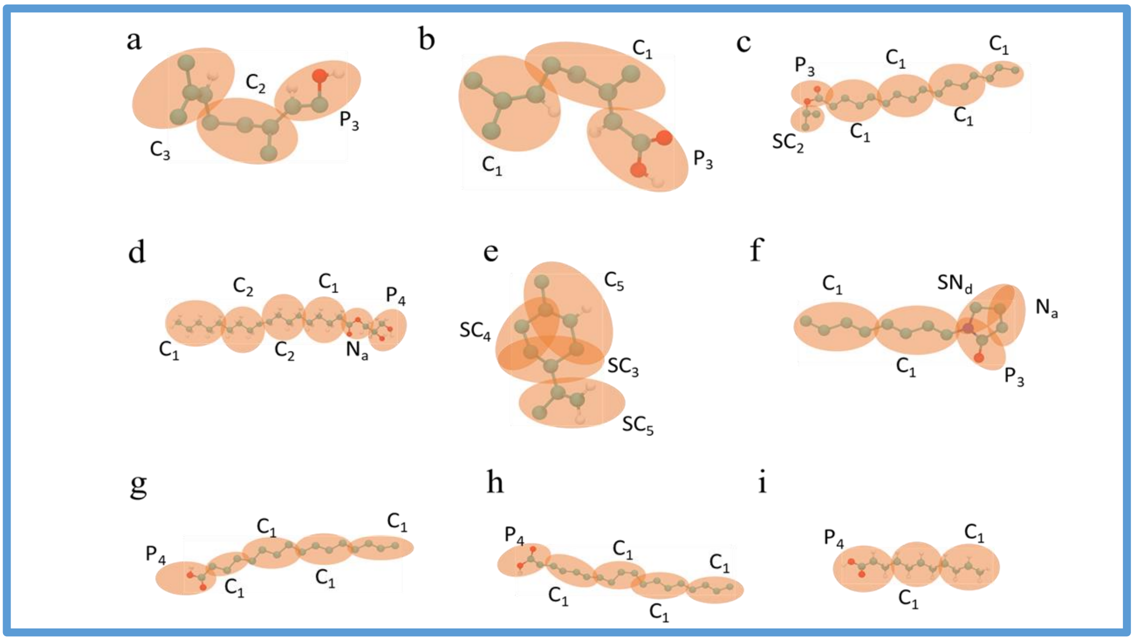

Figure S1. Mapping of the molecules into Martini beads (down). a) geraniol, b) geranic acid, c) isopropyl palmitate, d) monoolein, e) limonene, f) n-octyl pyrrolidone, g) palmitic acid, h) oleic acid and i) undecanoic acid. Images were created using VMD software.

The mapping of the atoms into beads was carried out based on MARTINI philosophy. Each bead consists of 3 or 4 atoms, and the bead types were assigned based on the polarity. Bonded parameters of molecules Geraniol, Geranic Acid, Isopropyl Palmitate, Monoolein, Limonene, N-octyl Pyrrolidone were developed by modifying the coarse grain (CG) bonding parameters till a reasonable match of the bonded distributions is obtained as compared to those obtained from atomistic simulations. Also, the bulk density of the CG simulation was compared to the atomistic simulation. All atom simulations, with atleast 300 molecules were carried out for 30 ns and the trajectories were sampled to obtain bond and angle distributions and the bulk density. CG simulations with atleast 300 molecules (number equal to those taken in all atom simulations) were run for 10 ns with a time step of 25 fs, from which the bond and angle distributions and bulk density were obtained. The parameterization of bond parameters was quite straight forward in structures without a ring, but was tricky with ring containing structures (limonene and N-octyl pyrrolidone). The bonded parameters for limonene were taken from those of tyrosine as given in the martini amino acids files [1, 2].

Figures S1-S4 show the comparison of bond and angle distributions obtained from CG (martini) simulations and all atom simulations. These are in reasonable agreement. It should

be kept in mind that in coarse graining, we may not get an exact match with atomistic distributions. Care should be taken that the bonds/angles do not get too rigid. Thus in a few cases (Ex: Fig S1 b1), it can be seen that the distributions match in mean position while the probability may not be as steep. These values gave us reasonable match with bulk density as reported in the main text. Table s1 gives the bonded parameters of the six molecules.

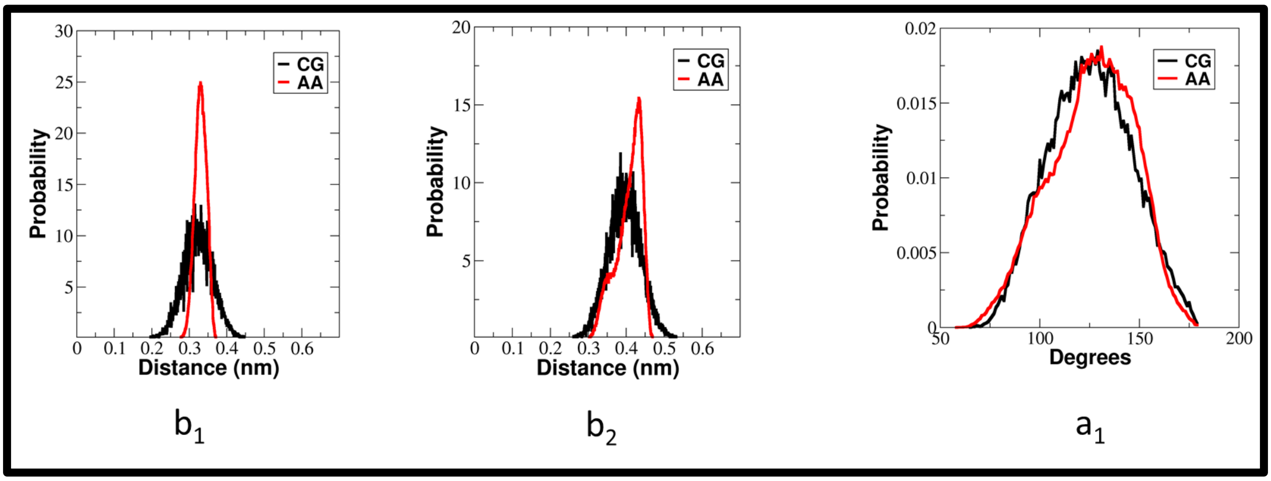

Figure S2. Bond and angle distributions for Geraniol.

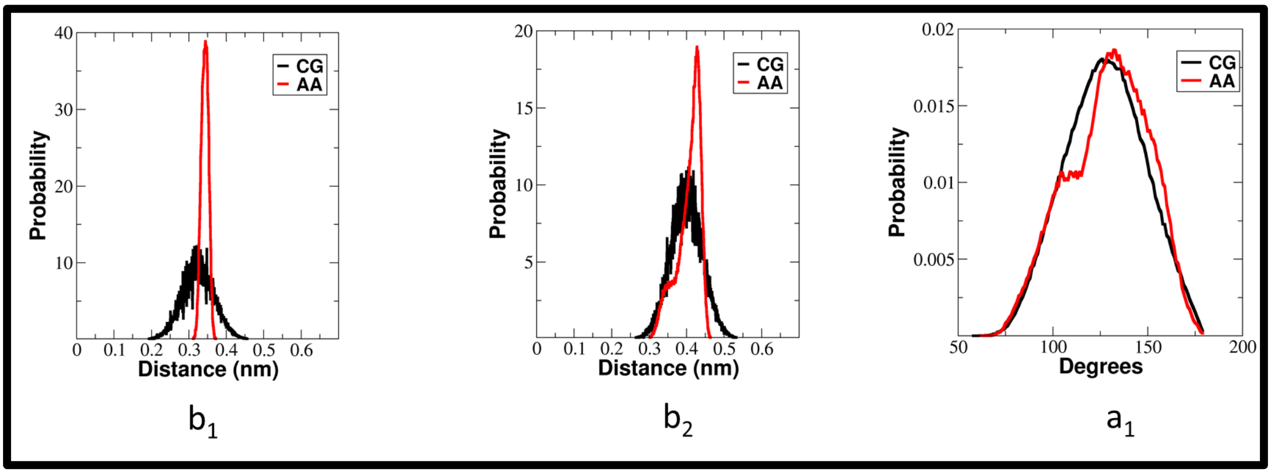

Figure S3. Bond and angle distributions for Geranic Acid.

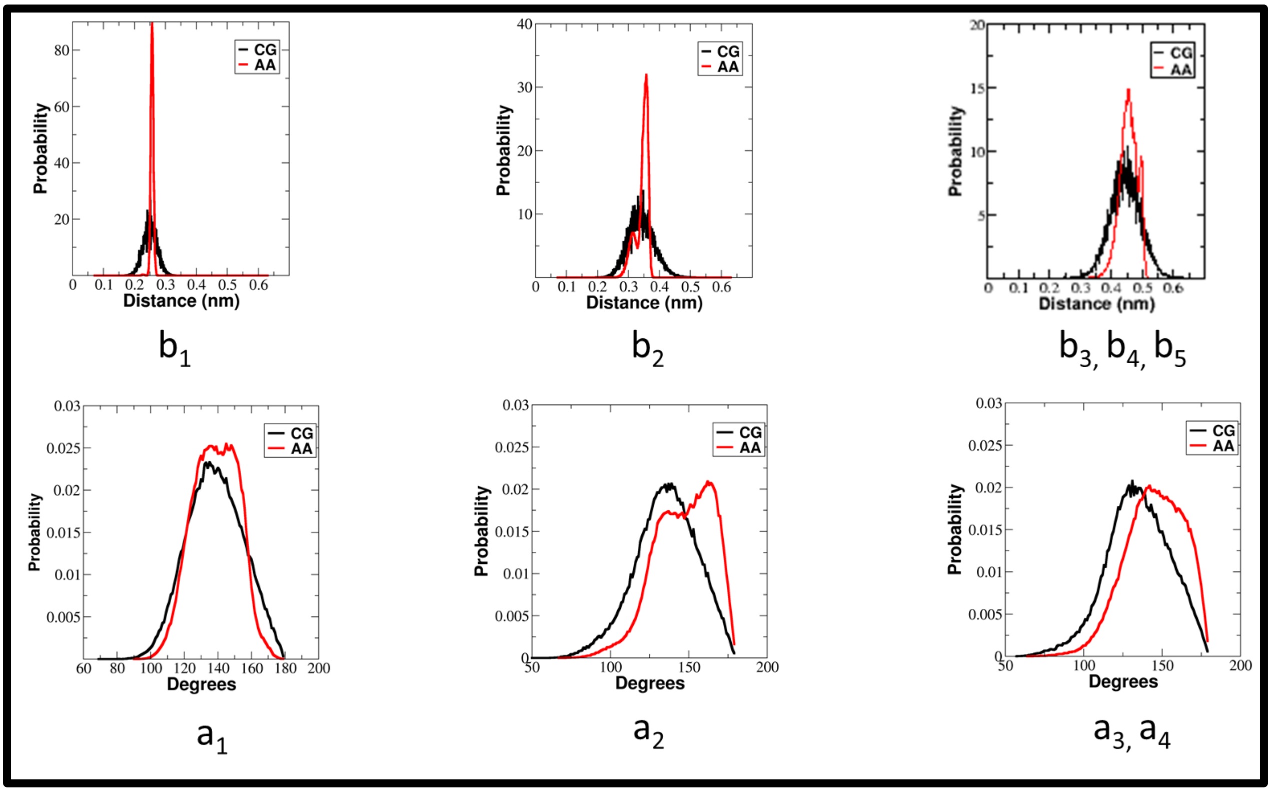

Figure S4. Bond and angle distributions for Isopropyl palmitate.

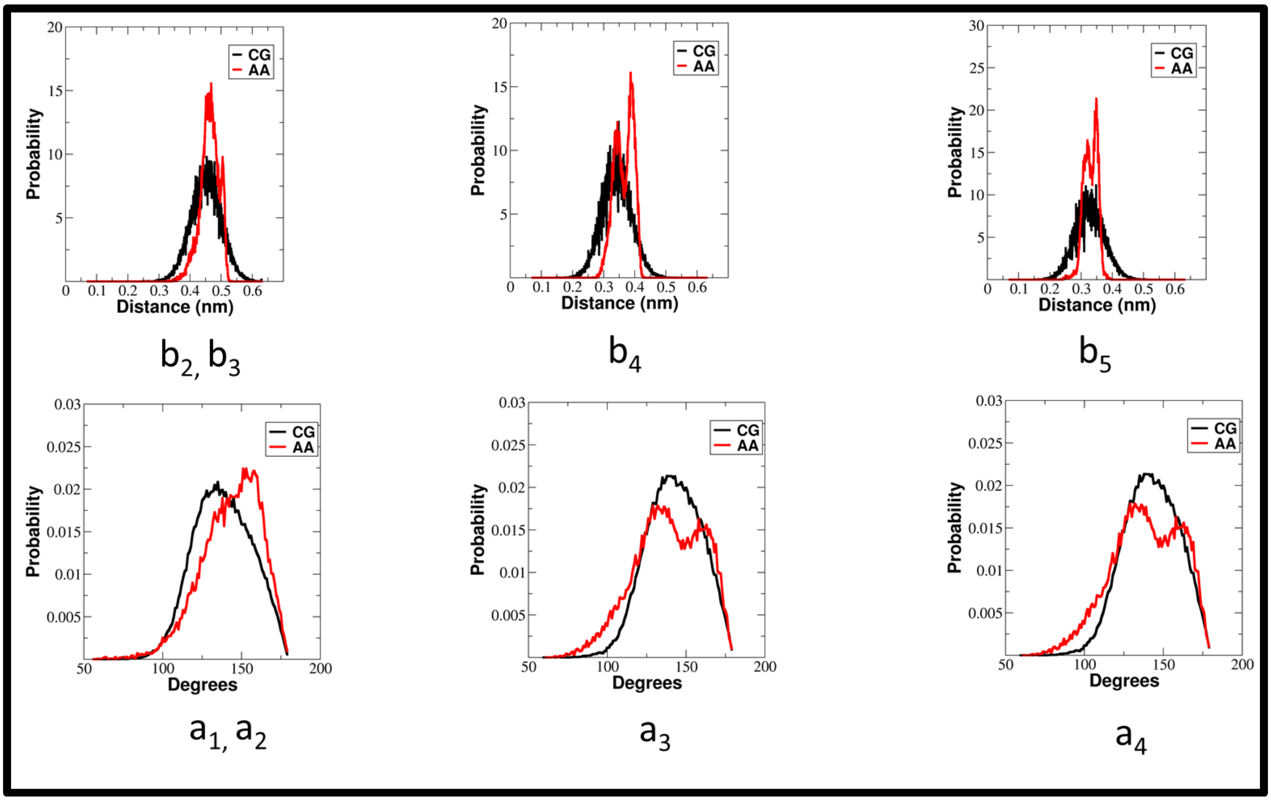

Figure S5. Bond and angle distributions for Monoolein

Table S2. Bonded parameters for molecules

Geraniol				
	Bead types	b_o (nm)	K_b (KJ/mol nm^2)	
b1	P3-C2	0.350	1750	
b2	C2-C3	0.420	1500	
		θ_o (deg)	K_θ (KJ/ mol)	
a1	P3-C2-C3	130	15	
Geranic Acid				
	Bead types	b_o (nm)	K_b (KJ/mol nm^2)	
b1	P3-C1	0.350	1500	
b2	C1-C1	0.400	1500	
		θ_o (deg)	K_θ (KJ/ mol)	
a1	P3-C1-C1	130	15	
Isopropyl Palmitate				
	Bead types	b_o (nm)	K_b (KJ/mol nm^2)	
b1	SC2-P3	0.260	5000	
b2	P3-C1	0.370	1250	
b3, b4, b5	C1-C1	0.470	1250	
		θ_o (deg)	K_θ (KJ/ mol)	
a1	SC2-P3-C1	138.56	25	
a2	P3-C1-C1	160.00	15	
a3, a4	C1-C1-C1	160.00	15	
Monoolein				
	Bead types	b_o (nm)	K_b (KJ/mol nm^2)	
b2, b3	C2-C2, C2-C1	0.475	1250	
b4	C1-Na	0.375	1250	
b5	Na-P4	0.375	1250	
		θ_o (deg)	K_θ (KJ/ mol)	
a1, a2	C1-C2-C2, C2-C2-C1	150	25	
a3	C2-C1-Na	140	15	
a4	C1-Na-P4	125	25	
Limonene				
	Bead types	b_o (nm)	K_b (KJ/mol nm^2)	
b1	SC5-SC3	0.320	5000	
b2,b3,b4	SC3-C5, SC3-SC4, C5-SC4	0.270	constraint	
		θ_o (deg)	K_θ (KJ/ mol)	
a1	SC5-SC3-C5	150	50	
a2	SC5-SC3-SC4	150	50	
		φ_o (deg)	K_φ (KJ/mol)	n
d1	SC5-SC5-SC4-SC3	0.00	50.0	2

Octyl Pyrrolidone			
	Bead types	b_o (nm)	K_b (KJ/mol nm^2)
b1	C1-C1	0.47	2000
b2	C1-P3	0.25	1750
b3	P3-Na	0.180	constraint
b4	P3-SNd	0.230	constraint
b5	Na-SNd	0.192	constraint
		θ_o (deg)	K_θ (KJ/ mol)
a1	C1-C1-P3	180	20

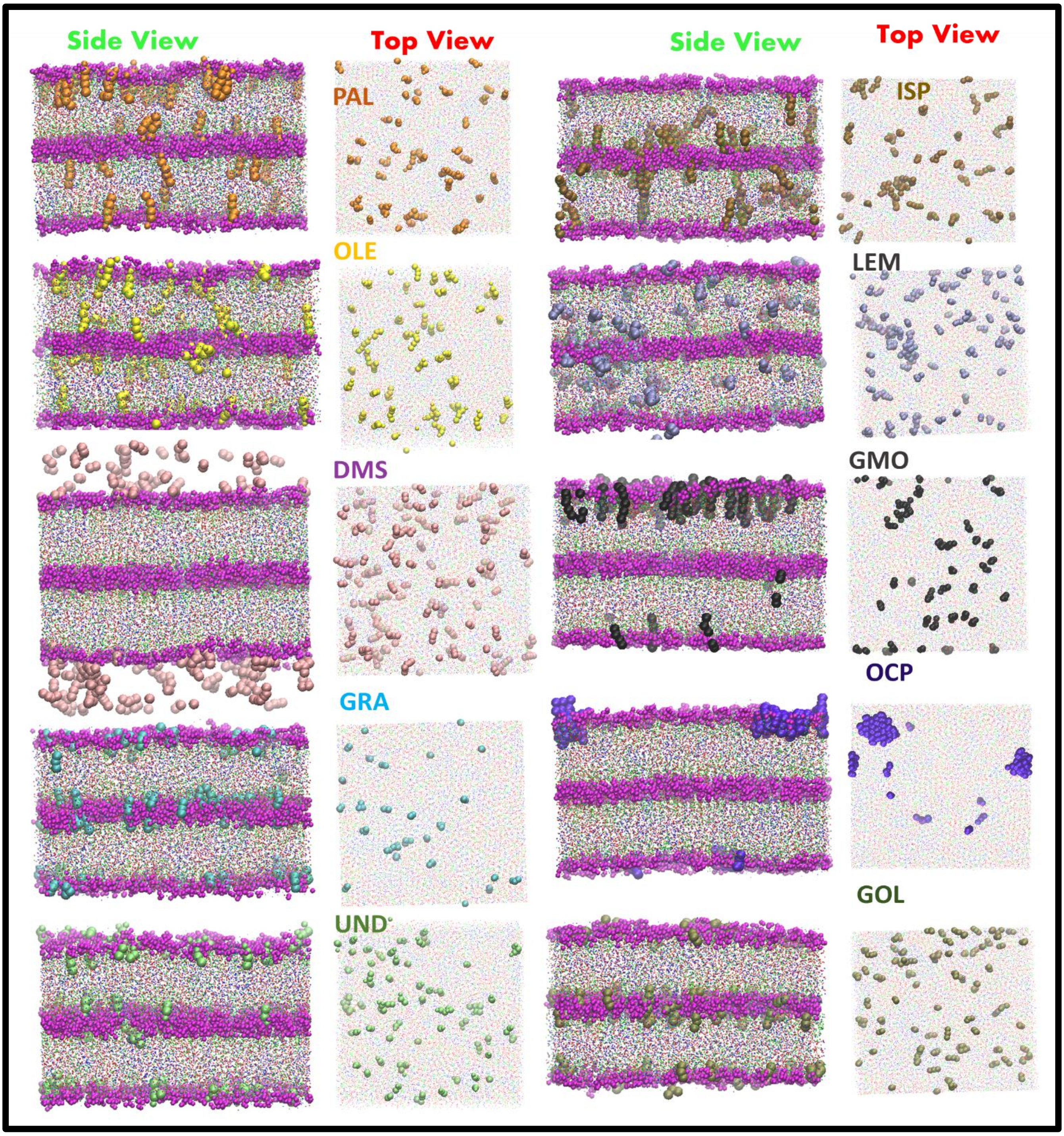

Figure S6. Snapshots of final configuration of skin double layer interacting with chemical permeation enhancers (at 3w/v concentration). Both side view (shows permeation of CPE's) and top view (shows dispersion or agglomeration of CPEs inside the layers) are shown. All systems were run for 3 μs. The skin lipid constituent CER, CHOL and FFA are shown (in point form of VMD software) in red, green and blue colors, respectively. The solvent (ethanol and water) are shown here. The permeation enhancers are shown in VDW form of VMD software.

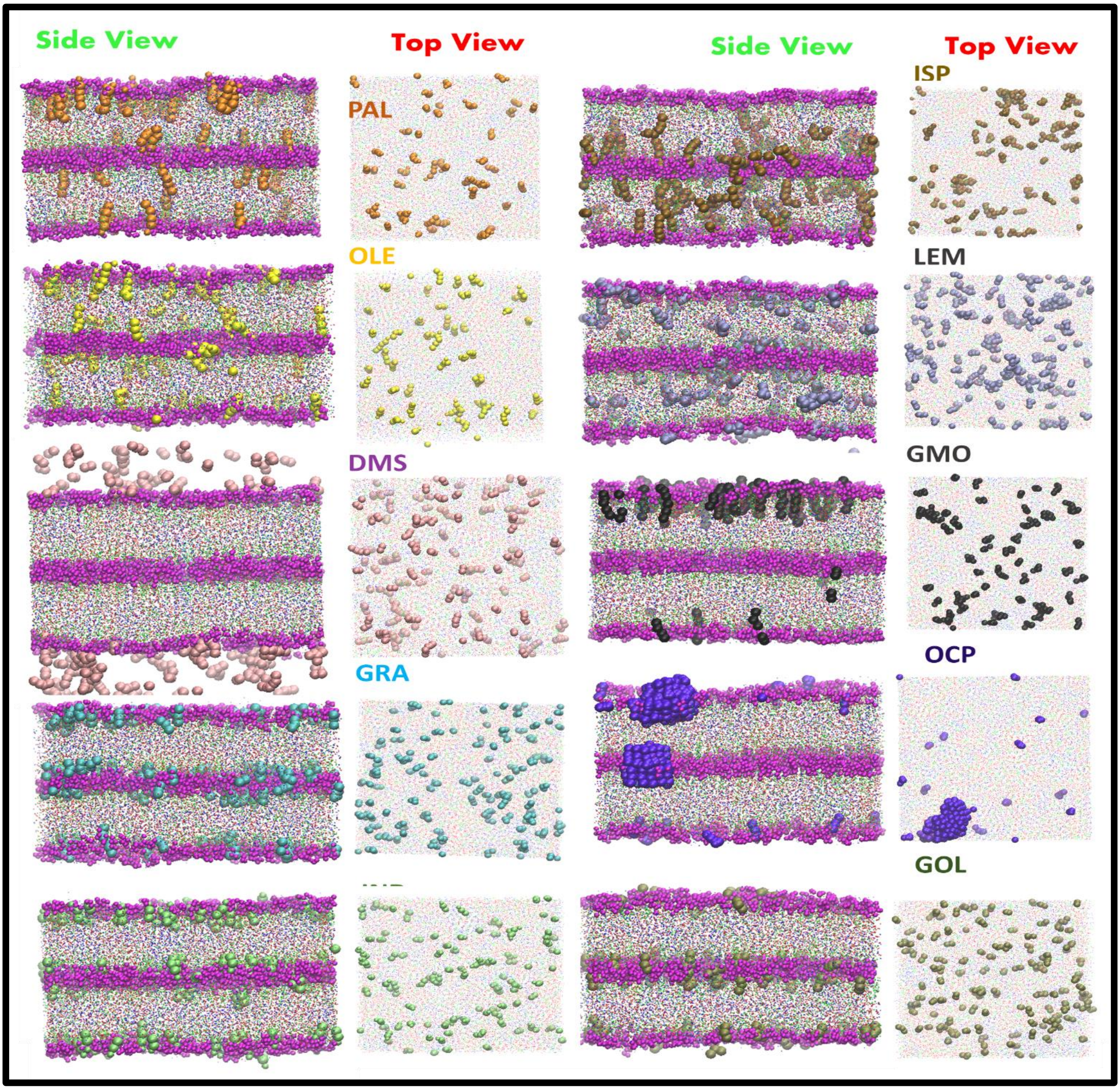

Figure S7. Snapshots of final configuration of skin double layer interacting with chemical permeation enhancers (at 5w/v concentration). Both side view (shows permeation of CPE's) and top view (shows dispersion or agglomeration of CPEs inside the layers) are shown. All systems were run for 3 μs. The skin lipid constituent CER, CHOL and FFA are shown (in point form of VMD software) in red, green and blue colors, respectively. The solvent (ethanol and water) are shown here. The permeation enhancers are shown in VDW form of VMD software.

S3. Radial distribution function of CPEs

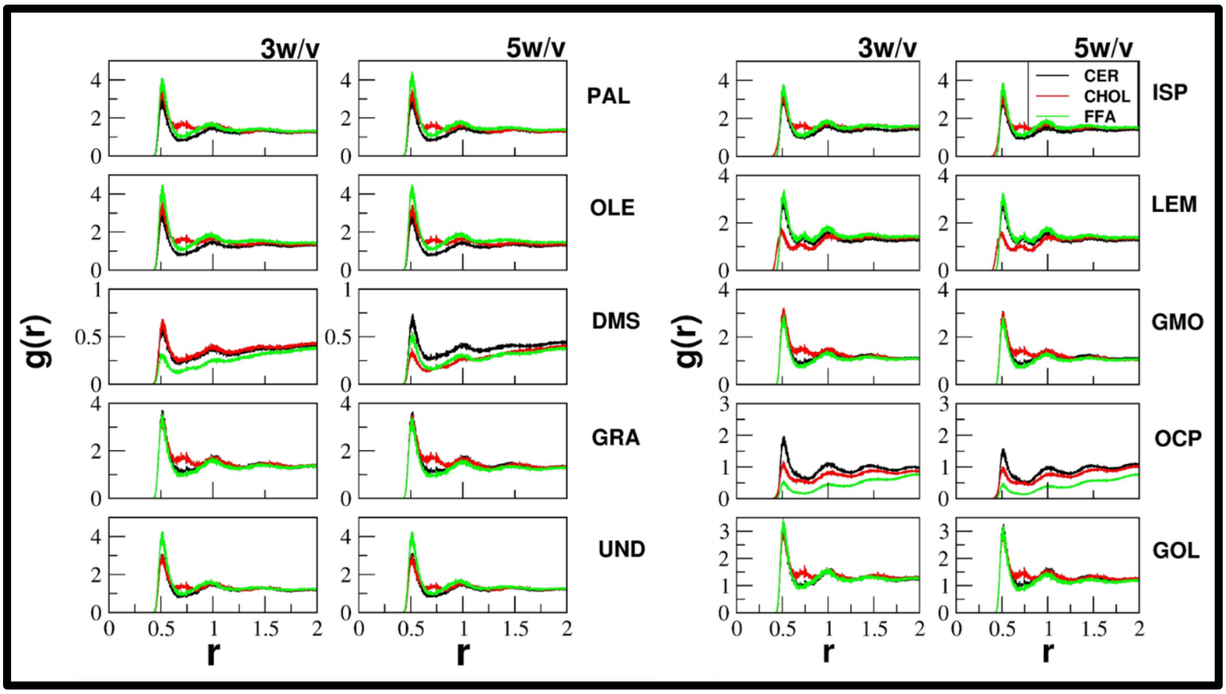

Figure S8. The radial distribution function g(r) of each permeation enhancer with skin lipid constituents (at 3 %w/v and 5 %w/v concentration), calculated in last 500 ns of production run. The peaks in g(r) profiles show the extent of the interaction between permeation enhancers and the particular lipid constituent.

S4. Mean Square displacement of CPEs

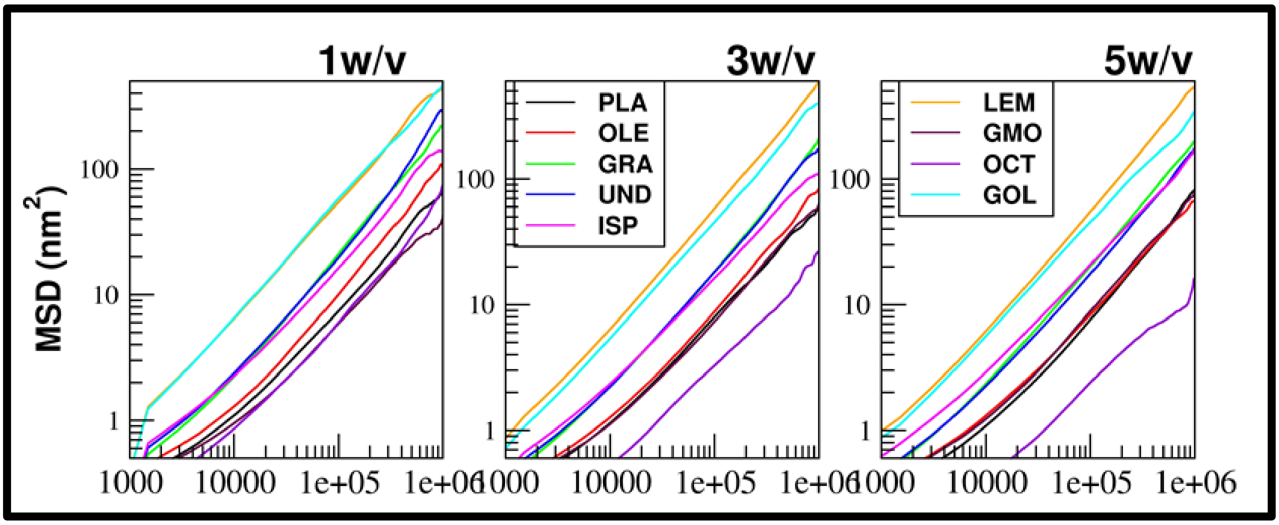

Figure S9. The mean square displacement of each permeation enhancers in skin lipid bilayer. The MSD was calculated in last 1 µs of production run.

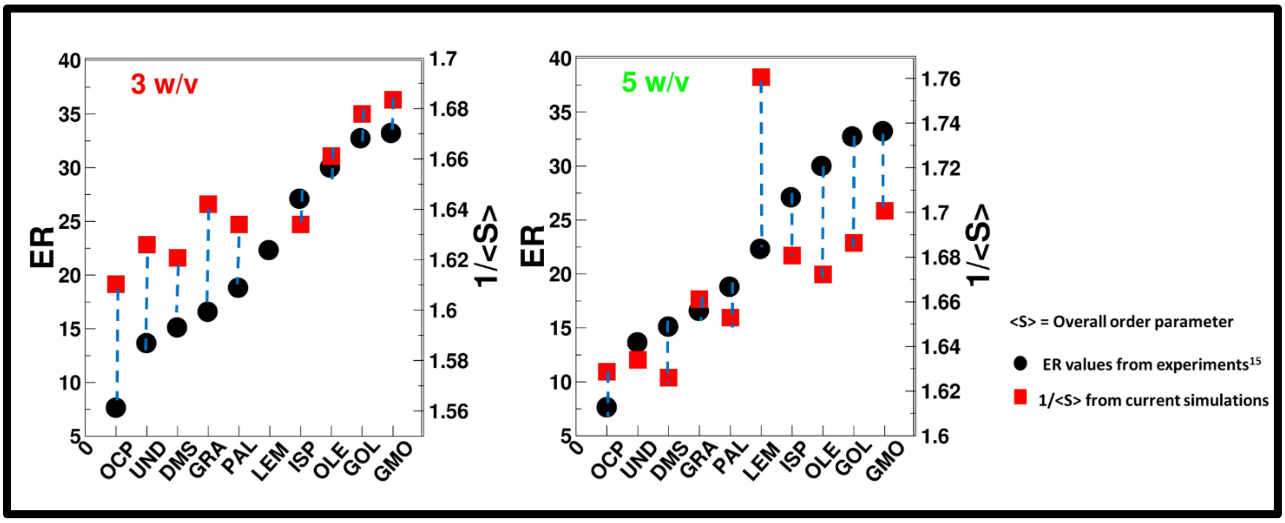

Figure S10. Comparison of experimental property enhancement ratio (ER) and the calculated property (1/<*S*>) from simulations for system 3%w/v and 5 %w/v. Both properties are plotted on different graphs due to the difference in the magnitude of each property.

References:

1. Monticelli, L., Kandasamy, S.K., Periole, X., Larson, R.G., Tieleman, D.P. and Marrink, S.J., 2008. The MARTINI coarse-grained force field: extension to proteins. Journal of chemical theory and computation, 4(5), pp.819-834

2. de Jong, D.H., Singh, G., Bennett, W.D., Arnarez, C., Wassenaar, T.A., Schäfer, L.V., Periole, X., Tieleman, D.P. and Marrink, S.J., 2012. Improved parameters for the martini coarse-grained protein force field. Journal of Chemical Theory and Computation, 9(1), pp.687-697.
